# Internet-Based Cognitive Behavioral Therapy for Children and Adolescents With Dental Anxiety: Open Trial

**DOI:** 10.2196/jmir.7803

**Published:** 2018-01-22

**Authors:** Shervin Shahnavaz, Erik Hedman-Lagerlöf, Tove Hasselblad, Lena Reuterskiöld, Viktor Kaldo, Göran Dahllöf

**Affiliations:** ^1^ Division of Pediatric Dentistry Department of Dental Medicine Karolinska Institutet Huddinge Sweden; ^2^ Department of Clinical Neuroscience Karolinska Institutet Stockholm Sweden; ^3^ Department of Psychology Stockholm University Stockholm Sweden; ^4^ Centre for Psychiatry Research Department of Clinical Neuroscience Karolinska Institutet Stockholm Sweden

**Keywords:** cognitive behavioral therapy, dental fear, dental phobia, dentistry, internet-based treatment, pediatric dentistry, psychology, self efficacy

## Abstract

**Background:**

Cognitive behavioral therapy (CBT) is an evidence-based method for treating specific phobias, but access to treatment is difficult, especially for children and adolescents with dental anxiety. Psychologist-guided Internet-based CBT (ICBT) may be an effective way of increasing accessibility while maintaining treatment effects.

**Objective:**

The aim of this study was to test the hypothesis that psychologist-guided ICBT improves school-aged children’s and adolescents’ ability to manage dental anxiety by (1) decreasing avoidance and affecting the phobia diagnosis and (2) decreasing the dental fear and increasing the target groups’ self-efficacy. The study also aimed to examine the feasibility and acceptability of this novel treatment.

**Methods:**

This was an open, uncontrolled trial with assessments at baseline, posttreatment, and the 1-year follow-up. The study enrolled and treated 18 participants. The primary outcome was level of avoidance behaviors, as measured by the picture-guided behavioral avoidance test (PG-BAT). The secondary outcome was a diagnostic evaluation with the parents conducted by a psychologist. The specific phobia section of the structured interview Kiddie-Schedule for Affective Disorders and Schizophrenia for School-Age Children-Present and Lifetime (K-SADS-PL) was used. Other outcome measures included level of dental anxiety and self-efficacy. The ICBT, which employed exposure therapy, comprised 12 modules of texts, animations, dentistry-related video clips, and an exercise package (including dental instruments). Participants accessed the treatment through an Internet-based treatment platform and received Web-based guidance from a psychologist. Treatment also included training at dental clinics. Feasibility and acceptability were assessed by measures of engagement, adherence, compliance, completed measures, patient and parent satisfaction scale, and staff acceptability.

**Results:**

The level of avoidance (according to the primary outcome measure PG-BAT) and dental anxiety decreased and self-efficacy increased significantly (*P*<.001), within-group effect sizes for both the primary outcome (Cohen d=1.5), and other outcomes were large in the range of 0.9 and 1.5. According to K-SADS-PL, 53% (8/15) of the participants were free from diagnosable dental anxiety at the 1-year follow-up. At the 1-year follow-up, improvements were maintained and clinically significant, with 60% (9/15) of participants who had been unable to manage intraoral injection of local anesthetics before ICBT reporting having accomplished this task at a dental clinic. The target group showed improvement in all the outcome measures. High levels of feasibility and acceptability were observed for the treatment.

**Conclusions:**

ICBT is a promising and feasible treatment for dental anxiety in children and adolescents. Integrating it into routine pediatric dental care would increase access to an effective psychological treatment. The results of this open trial must be replicated in controlled studies.

## Introduction

### Background

Dental fear and anxiety is defined as strong negative feelings associated with dental treatment or anticipation of dental treatment. Among children and adolescents, the prevalence of dental fear and anxiety is approximately 9% [1]. The Diagnostic and Statistical Manual of Mental Disorders-4^th^ edition (DSM-IV), often used in psychiatric or psychological research, classifies dental anxiety as a form of specific phobia and defines it as a persistent, irrational, and intense fear of a specific object or medical procedure in dentistry persisting for at least 6 months [2]. Dental anxiety often begins during childhood or adolescence. It leads to poor oral health manifesting as untreated caries, missing teeth, or periodontal problems [3] and can have other negative consequences such as a sense of embarrassment and reduced self-esteem [4].

The common methods for dealing with dental anxiety in pediatric dentistry are tell-show-do, sedation with midazolam, nitrous oxide sedation, and general anesthesia [5,6]. According to a recent systematic review of methods in pediatric dentistry [7], the evidence supporting these methods is low, and it is uncertain whether they reduce dental anxiety. This highlights the need for new, evidence-based psychological methods for treating dental anxiety in pediatric dentistry [8,9].

### Cognitive Behavioral Therapy

Cognitive behavioral therapy (CBT) is an evidence-based treatment method for psychiatric conditions such as specific phobias [10,11]. Main features of CBT include psychoeducation, coping techniques, cognitive restructuring, exposure, and homework exercises. CBT has been shown to be highly effective in adults with dental anxiety [12,13]. In a recently conducted randomized clinical trial, our research group showed that CBT has similarly large effects in children and adolescents [14]. Results from a qualitative study show that children experience increased feelings of safety and mastery and reduced fear in dental situations after receiving CBT [9,15]. However, accessibility to treatment is low. Children and parents face challenges that make it difficult to receive face-to-face CBT, such as constraints in time and availability, long distances to specialist pediatric dental clinics or lack of a psychologist at dental clinics. Thus, children with dental anxiety need better access to evidence-based psychological treatments.

### Internet-Based Cognitive Behavioral Therapy

Internet-based cognitive behavioral therapy (ICBT) is based on the same principles as conventional CBT, although ICBT is delivered over the Internet instead of in face-to-face sessions. ICBT has effect sizes comparable with face-to-face CBT and has been scientifically evaluated for many psychiatric conditions in both adults and children [16,17]. ICBT has shown promising results in treating specific phobias in children [18-20], is easier to deliver, and is more cost-effective than face-to-face CBT. The Internet-based version of CBT thus improves access to CBT among children and young individuals. An open study of self-help CBT resources (available in paper-based and on-line versions) for children with dental anxiety showed that CBT resources are a feasible and acceptable intervention for the reduction of dental anxiety in children in the age range of 9 to 16 years [21]. The self-help CBT resources mentioned above lack therapist guidance. The ICBT with therapist guidance (psychologist contact on weekly basis through a chat system) we implemented in this study is an Internet adapted version of a previously evaluated face-to-face CBT program (including therapy sessions with a clinical psychologist on weekly basis). The face-to-face treatment program has been evaluated in a randomized controlled trial (RCT) study [14]. We produced the treatment manual for the ICBT program based on the face-to-face CBT manual, which we developed further and adapted for the Internet.

The aim of this study was to test the hypothesis that psychologist-guided ICBT improves school-aged children’s and adolescents’ ability to manage dental anxiety by (1) decreasing avoidance and affecting the phobia diagnosis and (2) decreasing the dental fear and increasing the target groups’ self-efficacy. The study also aimed to examine the feasibility and acceptability of this novel treatment.

## Methods

### Design

This study has a single-group, open-trial design. We conducted assessments at baseline, posttreatment, and the 1-year follow-up. All participants and parents (one parent if there was only one primary caregiver) provided written informed consent.

To be able to have a larger recruitment base, we chose to include both children and teenagers in the study. As children needed to be able to read and understand the written text on the Internet platform, we chose 8 years as the minimum age. Age 7 or 8 is often the set starting age for these types of interventions. Some studies also have similar age ranges (7-13 or 7-14 years) [22,23]. The maximum age was chosen based on the need for self-determination and integrity that teenagers from the age of 15 years are granted in Sweden. If we would have included patients older than 15 years, then we would have to create two different log-ins in the Internet platform for the parent and the adolescents, which would make the treatment administration more complicated and would reduce the feasibility of the treatment. In the current intervention, participants and their parents had a common log-in and access to all modules throughout the course of treatment. The material on the Internet platform was common to all ages participating in the study, but the psychologist guiding the child tailored his or her messages to the child’s age.

The regional ethics review board in Stockholm approved the study (Daybook no: 2014/633-31/5).

### Participants and Recruitment

We recruited participants in two phases from August 2014 to February 2015. Our team contacted both private (only in Stockholm) and public dental clinics in Sweden by email and encouraged them to advertise the study in their waiting rooms (posters could be ordered or downloaded from the website). Interested parents applied through the website of the Department of Dental Medicine at Karolinska Institutet (the website provided visitors with brief information about the study and a list of primary inclusion criteria (items 1-5, Textbox 1). Textboxes 1 and 2 list the complete eligibility and exclusion criteria for participation in the study. A history of unsuccessful CBT for dental anxiety (during the past 3 years) suggests that this treatment might not be suitable. Therefore, we found it unethical to offer a treatment based on previous nonimprovement and excluded participants with earlier CBT experience. This is a standardized procedure in psychological treatment studies [24]

**Figure 1:**
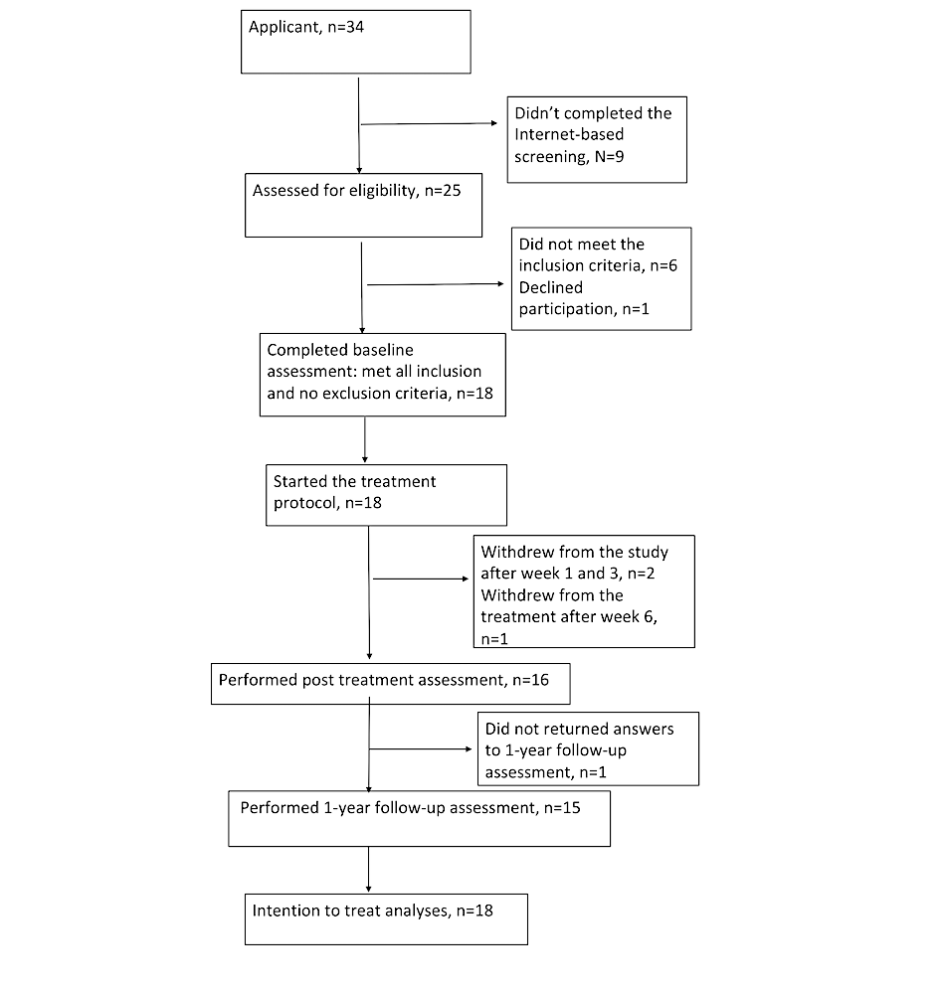
Flow of participants through the trial.

In all, 34 parents applied to participate in the study, and 18 children fulfilled all inclusion criteria and no exclusion criteria (we included a patient with attention deficit hyperactivity disorder incorrectly in the study; this patient received treatment and was kept in the analysis on the basis of intention-to-treat principle). Figure 1 shows the flow of participants through the trial.

The duration of dental fear before inclusion in the study was determined by directing the question “How many months has your child had his or her dental fear (including intraoral injection phobia)?” to the parent during the online screening (children and adolescents also received the question). The intensity of dental phobia was determined by 0 to 10 on a visual analog scale (VAS; no fear-strong fear) and the fear scale; Children’s Fear Survey Schedule-Dental Subscale (CFSS-DS). Intensity equal to or less than 3 on VAS and values less or equal to 31 on CFSS-DS evaluated by both child and parent were considered as too weak fear, which led to exclusion. Evaluation of duration and intensity of dental anxiety were also part of the diagnostic instrument K-SADS-PL, which were used during the telephone interview with the parents, evaluated by a psychologist.

**Inclusion criteria for participation in this study.**

1. The participant is in the age range of 8 to 15 years.
2. The participant had strong dental fear for at least 6 months before registering for the study.
3. The participant and parent have regular access to a computer and the Internet.
4. The participant is able to read and write in Swedish.
5. The participant has no current or planned psychological examination or treatment.
6. Participant and parents agree to participate in the research project.
7. The participant and parent have the time, opportunity, and motivation to work on and practice ICBT for 3 hours a week over 12 weeks.
8. Parents agree to book at least four visits at the dentist’s office during the 12 weeks of treatment.
9. Parents give their consent for the participant to be exposed to intraoral injection at the dentist if the child suffers from intraoral injection phobia, even if the child has no dental treatment needs.

**Exclusion criteria for participation in this study.**

1. Full scores on both the child and parent versions of the picture-guided behavioral avoidance test, which means that the child manages most of the procedures in dentistry.
2. A score of 31 or less on both the child and parent versions of the Children’s Fear Survey Schedule- Dental Subscale while, at the same time, not fulfilling the criteria for intraoral injection phobia.
3. Likely fulfillment of the criteria for a neurodevelopmental disorder according to the Development and Well-being Assessment and/or telephone interview by a psychologist.
4. Other psychiatric disorders such as severe depression, an eating disorder, or self-harm behavior that need treatment before dentistry-related specific phobia.
5. Stressful life experiences during the past 12 months, such as a difficult divorce in the family or somatic illness that the parent or psychologist sees as an obstacle to treatment.
6. A history of cognitive behavioral treatment for dental anxiety or needle phobia during the past 3 years.

Table 1 presents the sociodemographic and clinical characteristics of the participants in the intervention group.

### Primary Outcome Measure

The primary outcome measures were the child and parental versions of the picture-guided behavior avoidance test (PG-BAT). The PG-BAT is an analogous test to the behavior avoidance test (BAT), which is the recommended outcome measure for CBT studies of phobias [13,25,26]. The test is constructed by our research group and has been used in an earlier published study of face-to-face CBT for children and adolescents with dental phobia [14].

Our study chose the PG-BAT because it is adapted to pediatric dentistry and is self-reported, possible to conduct on Internet, in contrast to the BAT, which is administered by clinicians face-to-face. The PG-BAT consists of 17 hierarchically organized dental-clinical situations. During this online self-reported test, the participant views pictures of a child going through different dental procedures. The participant responds, using yes or no answers, whether he or she could manage the situation. The test comprises these situations: going into the dental treatment room, sitting in the treatment chair, fastening a paper bib around the neck, lowering back the chair, opening mouth, undergoing a clinical exam with mirror and dental probe, dropbox receiving topical anesthesia, receiving an injection of local anesthesia, undergoing drilling, and undergoing extraction. The same process applies for the parental version (parents assess the child’s ability to manage the dental procedures listed above).

**Table 1:** Patient and clinical characteristics (n=18).

Characteristic			Value
**Patient characteristics**			
	Age (years), mean (SD)		11 (2)
	Gender (female), n (%)		11 (61)
	Parent or sibling with dental fear, n (%)		3 (6)
	University education, mother, n (%)		7 (39)
	University education, father, n (%)		8 (47)
	One or both parents born in a country other than Sweden, n (%)		3 (17)
	Parents live together, n (%)		12 (67)
**Clinical characteristics**			
	Comorbidity^a^, n (%)		6 (30)
	Duration of dental anxiety (years), mean (SD)		4 (3)
	Intraoral injection as main fear, n (%)		14 (78)
	**Main reason for onset of fear**		
		Negative experiences in dentistry, n (%)	3 (17)
		Negative experiences in health care, n (%)	11 (61)
		Model learning through parent or sibling, n (%)	2 (11)
		Do not know the reason, n (%)	2 (11)
	Experience of nitrous oxide, midazolam, or general anesthesia before ICBT^b^, n (%)		11 (60)
	Referred to pediatric dental clinic, n (%)		8 (44)

^a^Comorbidity diagnoses were phobia of dogs, wasps, or blood, as well as attention deficit hyperactivity disorder and language disorder.

^b^ICBT: Internet-based cognitive behavioral therapy.

The score for each child was the sum of positive responses to these items, from 0 (not entering the dentist’s room) to 17 (managing all activities including dental extraction), with 1 point for each stage until the point where the child discontinued the test. According to data (N=26) generated in an ongoing study, both the child and parental versions of this test have shown good internal reliability (with a Cronbach alpha of. 88 and .86, respectively) and validity (significant positive association between the instruments and a clinician-conducted face-to-face BAT; *r*=.68, N=36; *P*<.001 for the child version and *r*=.75, N=37; *P*<.001 for the parental version).

### Secondary Outcome Measure

*Diagnosis of dental phobia* assessed the presence of dentistry-related specific phobia as measured by the phobic disorders supplement included in K-SADS-PL. K-SADS-PL is a semistructured diagnostic interview guide that generates reliable and valid psychiatric diagnoses [27]. We conducted interviews by telephone with one parent of the participants. All other outcome measures were self- or parent-reported and administered online.

### Other Outcome Measures

*CFSS-DS* child and parental versions, which consist of 15 items measuring the degree of fear associated with various situations in dental and medical care and with interactions with people unfamiliar to the child (scale 1-5, from no fear to high fear). The CFSS-DS for the Swedish version of the test has high test-retest reliability and validity [28].

*Self-Efficacy Questionnaire for Specific Phobias* (SEQ-SP), which consists of 14 questions assessing the level of self-efficacy (a 5-point scale, with the endpoints 1=low self-efficacy and 5=high self-efficacy). We used a version we translated to Swedish and adapted to dentistry. Flatt and King [29] provide evidence for the reliability and validity of the SEQ-SP.

*Children’s negative cognitions in dentistry* (CNCD), as measured using a scale constructed by our research team and inspired by a scale used in an earlier study of CBT for adults in dentistry [13]. Our scale contains 5 items (a VAS with both numbers and happy and sad faces and the endpoints 0=not having negative thoughts and 10=having negative thoughts). It asks children about both the presence and strength of five negative thoughts that are common in dentistry: uncontrollability, distrust of dentists, unpredictability, dangerousness, and pain related to dentistry [30,31]. We assessed the internal consistency of this scale by analyzing a sample from this study and a sample from another ongoing study; reliability is good (Cronbach alpha coefficient of .89, n=26).

*Parental Self-Efficacy Questionnaire for Dental Anxiety* (*P*-SEQ-DA), which is another measure constructed by our research team according to guidelines for constructing self-efficacy scales [32]. It comprises 12 items asking parents to evaluate their ability to support their children in dental situations (with the endpoints 0=no parental self-efficacy and 100=very high parental self-efficacy). A sample from this study showed the *P*-SEQ-DA to have good internal consistency (Cronbach alpha reliability coefficient of .89, n=26).

*Injection phobia scale for children* (IPSC), which measures changes in children’s degree of anxiety related to injection, consists of 18 items measuring the degree of fear associated with various situations associated to injection (scale 1-5, from no fear to high fear). The test has good reliability and validity [33,34].

In addition, participants and their parents responded to online questionnaires concerning the qualitative aspects of dental anxiety and ICBT with open answers, multiple choice questions, and VASs. We included questions evaluating the degree of satisfaction with the treatment. These questionnaires were delivered to the participants immediately after the treatment (week 13).

### Procedure

The recruitment process began with an online screening comprising questionnaires about informed consent for primary caregivers and children and background information and inclusion and exclusion criteria for participation, as well as the CFSS-DS (child and parental), PG-BAT (child and parental), CNCD, and IPSC.

In the second step, parents received access to the Development and Well-Being Assessment (DAWBA) on the Internet and a personalized password. The DAWBA is a package of questionnaires and rating scales designed to generate the 10th revision of the International Statistical Classification of Diseases and Related Health Problems and DSM-IV psychiatric diagnoses for children in the age range of 5 to 17 years [35,36].

In the third step, parents were administered the K-SADS-PL over the phone to determine whether the child met the inclusion criteria for dental anxiety (including intraoral injection phobia) and other phobias (including general injection phobia, eg, vaccination). On the basis of the DAWBA and K-SADS-PL, a clinical psychologist determined if dental anxiety was the primary diagnosis and whether the participant met the inclusion criteria. If the child was over the age of 11 years, the psychologist conducted a short telephone interview with the child to ensure that the child had read the information about the project and was willing to participate. We conducted assessments before treatment (baseline), after treatment (12 weeks after the start of treatment), and at a 1-year follow-up. All assessments used online questionnaires except for the parent interview conducted with K-SADS-PL and questions about dental health staff and their acceptance of the treatment. These were administered by telephone interviews.

### Intervention

The treatment manual comprised 12 modules (modules are distinct but interrelated units that ICBT is built upon) of guiding text (32,000 words), 18 worksheets, and 10 informational documents. Table 2 shows the content of modules. We wrote the manual in Swedish, developing it and adapting it to the Internet from a face-to-face CBT manual [14]. Four experienced clinical psychologists and one specialist in pediatric dentistry (coauthors) read the manual and gave continuous feedback during its development. Participants accessed treatment modules through a specially designed participant-secure platform for Internet-based psychological treatments.

**Table 2:** Contents of the modules.

Modules		Content	
**Intro (Modules 1-2)**		
	1-2	Coach psychoeducation; practical arrangements; home assignment; how to guide a child to elicit and reinforce behavioral change; rewarding strategies; and enhancing the child's self-efficacy
**Exposure (Modules 3-11)**		
	3	Behavioral analyses; child psychoeducation and treatment rationale; goal setting
	4	Constructing an exposure list and beginning exposure
	5	Continued exposure (films and training package) and controlled breathing
	6	Dentistry-related communication training; preparation for dental visit
	7	Evaluation of dental visit; cognitive restructuring
	8	Evaluation of ICBT^a^ (so far); evaluation of exposure or treatment at a dental clinic; relaxation techniques
	9	Pain and pain management education; fear, thoughts, and pain; focus shift and acceptance training
	10	Problem solving and mindfulness training
	11	Repetition; strategies for maintaining change and relapse prevention; letter to yourself
**Diploma (Module 12)**		
	12	Relapse prevention plan; enhance your self-efficacy; diploma

^a^ICBT: Internet-based cognitive behavioral therapy.

#### Treatment Introduction

The first two modules targeted the coach, who was a parent or other person significant to the child and accepted to take primary responsibility for assisting the child during the treatment. From module 3 onward, the text addressed the children directly. Each module consisted of a number of tasks (answering questions and filling out worksheets) that the participant had to complete before moving on to the next module.

**Figure 2:**
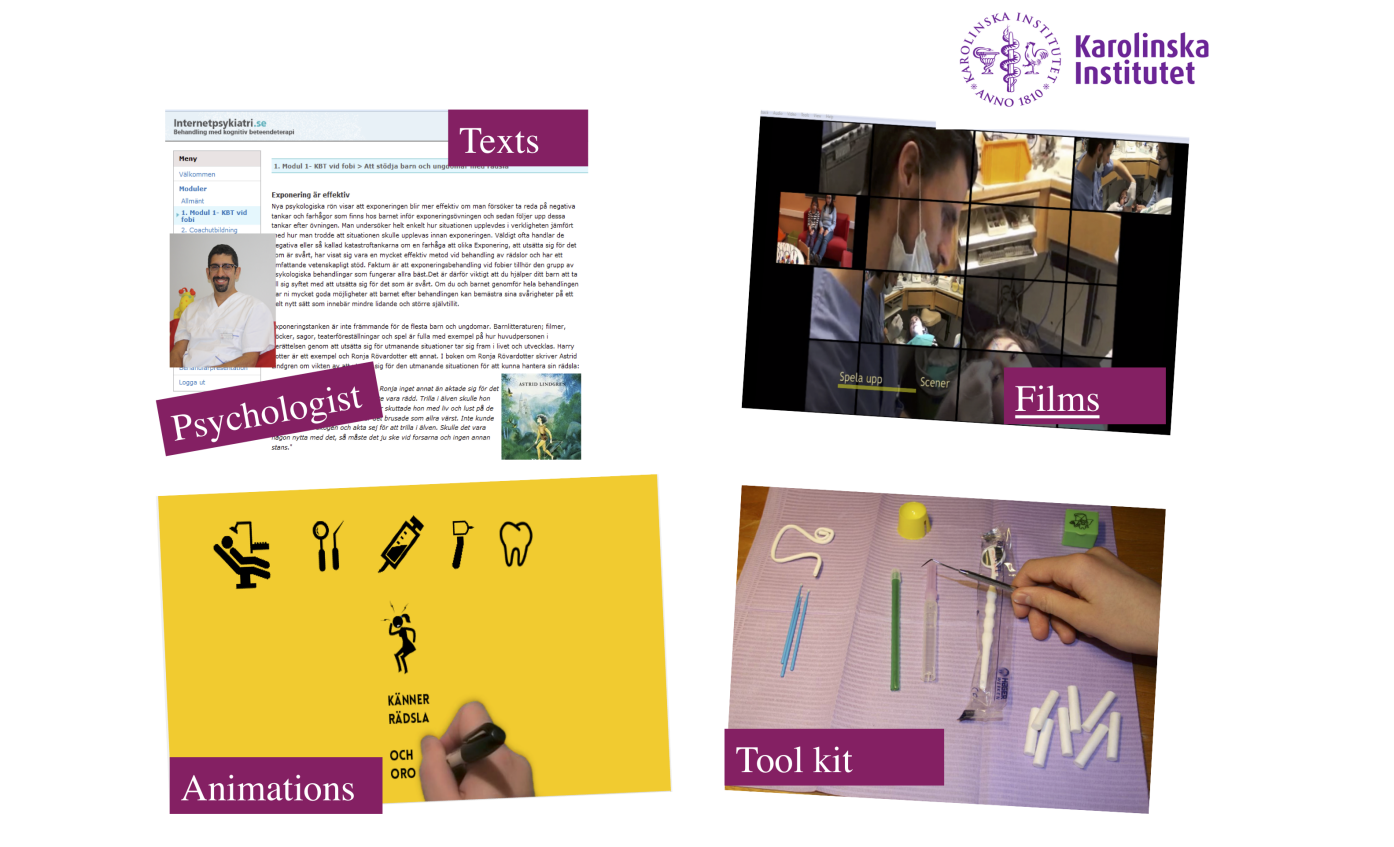
The components of the Internet-based treatment.

Figure 2 illustrates the components of the treatment. Early in treatment (module 2), we asked the coach to book a planning meeting (15 min) and a minimum of three dental appointments (30 min each) for exposure practice and dental care, to occur sometime between 6 weeks into the treatment and the end of treatment at 12 weeks. During treatment, the coach had access to an information sheet, available on the Internet platform that described the Internet treatment, basic aspects and rules of CBT, and the role of dental professionals during the Internet treatment. We asked coaches to send the information sheet to the dental clinics at which they had booked appointments. The dental care teams determined whether a dentist, dental hygienist, or dental assistant would take the main responsibility for exposure exercises at the dental clinic. The coach and children, together with their online psychologist, drafted suggestions concerning suitable exposure exercises that they then brought to the dental office for discussion.

#### Psychologist Contact

Each participant had a personal psychologist whom we introduced to the participant with a welcome letter at the beginning of treatment. Participants had continuous contact with their psychologist via a messaging system on the Internet platform. A psychologist guided the participants and their coaches during the 12 weeks of treatment. Two licensed psychologists provided this treatment. Both had a CBT qualification and experience in delivering CBT in pediatric dentistry. To increase treatment adherence and therapist competence, the psychologist with greater experience (8 years of experience as a therapist in pediatric dentistry) supervised the other psychologist on a weekly basis.

#### Homework

Each module ended with homework that contained both knowledge questions based on texts in the modules, as well as practical exercises such as exposures; registrations of, for example, negative thoughts related to dental care; relaxation exercises; and mindfulness. Exposure to dentistry-related video clips and audio files began from module 3. A practice package consisting of dental tools such as a dentist’s mirror, dental probe, topical anesthetic, and cannula were sent home to the coach together with detailed instructions for use and safety (tool kit in Figure 2). Parents and children had to complete the assigned homework after each session before they were allowed to progress to the next module. The psychologist sent a message to the participant once a week. Messages consisted of feedback on homework assignment and answers to questions parents or children raised. If the assignments were not completed, reminders were sent to the participant. Exposures at the dental office started after module 6. The psychologists would provide feedback and support on homework assignments to participants within 36 hours on weekdays.

#### Relapse Prevention and Diploma

The treatment was ended by repetition, strategies for maintaining change, and relapse prevention. The participant has the possibility to print a diploma accessible in module 12.

The treatment manual for this study is available in Swedish and English and can be obtained by contacting the corresponding author.

### Statistical Analyses

On the basis of an earlier study of CBT for pediatric dental anxiety [14], we expected a within-group Cohen *d* effect size of 0.80, which would require a sample size of 15 to achieve a power of 80% using a one-tailed test and alpha at .05.

The statistical analyses used Statistical package for the Social Sciences (SPSS) version 22 (IBM Corp). We analyzed data for the primary outcome measure (the PG-BAT, child and parental versions) and the other continuous measures (the CFSS-DS, child and parental versions; SEQ-SP; P-SEQ-DA; CNCS; and IPSC) using repeated measures analysis of variance. We also performed a separate sensitivity analysis for the primary outcome measure by putting missing data at the posttreatment and 1-year follow-up measurements (3 participants) with data from baseline assessments (assuming no change for these participants). Paired sample *t* tests compared continuous outcome measures from baseline to posttreatment, baseline to the 1-year follow-up, and posttreatment to the 1-year follow-up. Cochran Q test determined whether there were differences in the dichotomous dependent variables over time (from baseline to posttreatment to the 1-year follow-up). McNemar test explored if there were differences in the dichotomous dependent variables between two related groups (baseline to posttreatment, baseline to the 1-year follow-up, and posttreatment to the 1-year follow-up). Cohen *d*, based on pooled standard deviations, determined the effect sizes [37]. We evaluated clinically significant improvement on the basis of the primary outcome (level of avoidance behavior, as measured by the PG-BAT), setting a value of 12 or above as the cut-off value for clinically significant change. Values above 12 meant that the participant managed injection with local anesthesia, which is a necessary task in dentistry. Thus, we dichotomized the PG-BAT values based on a cut-off value of 12. This definition has been used in an earlier published study of face-to-face CBT for children and adolescents with dental phobia [14].

We also reported parents’ responses to an online question, which asked if the participants had undergone and managed intraoral injection at a dental clinic after the ICBT.

Engagement, adherence, and compliance were used as a measure of feasibility: Engagement is the number of participants who began a module. Adherence is the number of participants who completed at least 75% of the modules (9/12 modules), and compliance is the number who completed the treatment program (all 12 modules). We considered a treatment module as complete when the participants had completed the home assignment and returned it to the psychologist on the Internet platform.

## Results

### Attrition and Dropout

Of 18 participants who began treatment, 2 discontinued participation in the study (Figure 1). In one case, the coach (mother) found the texts and home assignments too difficult to understand and manage. She declined participation after completing module 2. In the other case, the coach only performed the first assignment (module 1) and did not respond to reminders for continued participation. Another parent and her child dropped out after performing module 6. The parent stated that this was because of a crisis in their life situation and other priorities. However, the parent agreed to and partly completed the assessments at the posttreatment measurement, but they were not available for the 1-year follow-up.

### Clinician Support and Adverse Effect

The average total clinician time per participant (including emails and telephone calls) was 5.4 hours (SD 2.3) which is approximately 30 min per week for each participant. We also asked participants and their parents about any negative or adverse effects of ICBT with a question in the online questionnaire they completed at the posttreatment assessment. None reported any negative effects.

### Primary Outcome

Statistically significant changes in children’s self-perceived ability to manage dental care procedures according to the PG-BAT, child version (*F*_2,28_=14.1,  *P*<.001) and in parental perceptions of the child’s ability to manage dental care (not avoiding dental care), as measured by the PG-BAT, parental version (*F*_2,28_=21.4,  *P*<.001) occurred. There was a significant within-group improvement in both the child and the parental versions of the PG-BAT from baseline to posttreatment and from baseline to the 1-year follow-up (Table 3). No significant changes from posttreatment to the 1-year follow-up occurred. The sensitivity analyses showed no difference in significance levels from the original tests, which were missing data for three participants.

### Secondary Outcome (Clinician Administered)

In the psychologist-administered telephone interviews with the parents, which used the K-SADS-PL (specific phobia section), there was a significant reduction over time in the proportion of participants with diagnosis dental anxiety (*P*=.001). We also found a significant reduction in the proportion of participants with a diagnosis of dental anxiety from baseline to posttreatment (*P*=.02) and from baseline to follow-up (*P*=.01). After treatment, 50% (7 /14) of participants no longer met the diagnostic criteria for dental anxiety and at the 1-year follow-up, 53% (8 /15). Corresponding reductions over time in injection phobia in the health care context (eg, vaccination) were also significant (*P*=.02). The proportion of participants with health care injection phobia reduced significantly from baseline to posttreatment (*P*=.008) but not from baseline to the 1-year-follow up (*P*=.06).

**Table 3:** Efficacy of cognitive behavioral therapy for children and adolescents with dental anxiety. Pre and post treatment and 1-year follow-up measures; *P* and *t* values; and effect sizes (according to Cohen *d*) and CIs.

Measures	Pre^a^ (N=16) Mean (SD^b^)	Post^c^ (N=16) Mean (SD)	Follow-up^d^ (N=15) Mean (SD)	Prepost			Prefollow-up		
				*P* value	*t* value	Effect sizes (95% CI)	*P* value	*t* value	Effect sizes (95% CI)
PG-BAT^e^ child version	10.5 (1.0)	13.9 (2.9)	13.7 (2.8)	<.001	5.5	1.5 (0.7-2.3)	.001	4.1	1.4 (0.3-2.6)
PG-BAT parental version	9.3 (2.8)	13.7 (3)	13.7 (2.9)	<.001	4.9	1.5 (0.6-2.5)	<.001	4.8	1.6 (0.5-2.6)
CFSS-DS^f^ child version	32.9 (10.2)	24.1 (6.8)	23.8 (6.4)	<.001	6.3	1.0 (0.5-1.6)	.006	3.2	1.1 (0.2-1.9)
CFSS-DS parental version	35.4 (10.1)	24.1 (6.3)	25.0 (4.8)	<.001	5.7	1.3 (0.7-2.0)	.001	4.5	1.3 (0.6-2.0)
SEQ-SP^g^	27.8 (8.3)	44.6 (6.9)	44.1 (11.1)	<.001	6.2	2.2 (0.8-3.6)	.001	4.3	1.66 (0.4-2.9)
P-SEQ-DA^h^	107.9 (13.3)	124.7 (7.2)	118.7 (11.6)	<.001	6.5	1.6 (0.9-2.2)	.02	2.6	0.9 (0.0-1.7)
CNCD^i^	24.0 (12.6)	8.0 (9.0)	11.4 (10.1)	<.001	5.4	1.7 (0.6-2.9)	.001	4.8	1.1 (0.5-1.8)
IPSC^j^	44.4 (14.3)	33.3 (10.9)	35.9 (12.1)	.001	4.05	0.9 (0.4-1.4)	.08	1.9	0.6 (−.05 to 1.3)

^a^Baseline measurement.

^b^SD: standard deviation.

^c^Posttreatment measurement (after 12 weeks of treatment).

^d^1-year follow-up (1 year after posttreatment).

^e^PG-BAT: picture-guided behavior avoidance test.

^f^CFSS-DS: Children's Fear Survey Schedule-Dental Subscale.

^g^SEQ-SP: Self-Efficacy Questionnaire for Specific Phobias.

^h^P-SEQ-DA: Parental Self-Efficacy Questionnaire for Dental Anxiety.

^i^CNCD: children’s negative cognitions in dentistry.

^j^IPSC: injection phobia scale for children.

### Other Outcomes (Self-Reported and Parent Reported)

There were significant changes in the participant’s self-perceived level of fear according to the CFSS-DS, child version (*F*_2,28_=10.1,  *P*<.001) and parental perceptions of the participant’s level of fear according to the CFSS-DS-P (*F*_2,28_=18.6,  *P*<.001). Similar results occurred in the child’s or adolescent’s level of self-efficacy, SEQ-SP (*F*_2,28_=19.4,  *P*<.001); parental self-efficacy, SEQ-DA-P (*F*_2,28_=12.6, *P*<.001); the CNCD (*F*_2,28_=19.4, *P*<.001); and for the general fear of injection (*F*_2,28_=6.9, *P*=.01). There was a statistically significant within-group improvement from baseline to posttreatment and from baseline to the 1-year follow-up for all the continuous outcome measures except for the injection phobia scale, where the significant changes from baseline to posttreatment were not maintained at the 1-year follow-up (Table 3).

### Effect Sizes

As Table 3 shows, large within-group effect sizes between baseline and posttreatment measurements and from baseline to the 1-year follow-up measurements occurred for both the primary measurements, level of avoidance (the PG-BAT), and other continuous measurements.

### Clinically Significant Improvement

There was a significant difference in the proportion of participants who managed to pass the cut-off value over time (baseline to post to 1-year follow-up) for both child- and parent-reported PG-BATs (*P*=.001). At the posttreatment measurement, 56% (9/16) of parents and children reported that the participant could manage an intraoral injection. The corresponding proportion was 60% (9/15) at the 1-year follow-up. At baseline, all participants and their parents had reported that the participant could not manage local anesthesia. The improvement from baseline to posttreatment (*P*=.008 for children and *P*=.002 for parents) and from baseline to 1-year follow-up (*P*=.008 for both) was significant for both the child or adolescent and parental versions of the PG-BAT. Moreover, agreeing with the dichotomized results of the PG-BAT above, 60% (9/15) of parents reported at the 1-year follow-up that their child had managed intraoral injection at the dental clinic.

### Feasibility and Acceptability

Measures of feasibility were engagement=18/18, adherence=14/18, and compliance=5/18. Children and their coaches completed, on average, 9.2 (SD 3.3) of the 12 treatment modules. Moreover, the completed measure (at posttreatment) rate was 90% (16/18). Measure of acceptance, that is, the average level of satisfaction with the treatment at posttreatment (from 1=quite dissatisfied to 4=very satisfied) for the participants was 3.3 (SD 0.6) and for the parents, 3.1 (SD 0.4). There were 15 children and 15 parents who were mostly satisfied or very satisfied (3 and 4 on the scale), whereas 1 child and 1 parent were indifferent (2 on the scale). Some parents and children, however, requested less extensive texts with fewer repetitions in the treatment manual. For all 15 children, the parents were able to book and attend at least three appointments at the dental clinic during the 12 weeks of ICBT. Participants visited a dental assistant, a hygienist, or a dentist during these visits.

Parents did not report (in the telephone interview at posttreatment) any major problems in terms of the dental personnel’s acceptance of the Internet treatment. Parents reported that most of the dental professionals who met the child or adolescent during ICBT accepted the Internet treatment and showed interest in the treatment, meaning that they followed the information sheet that provided instructions for the staff. In two cases, however, coaches reported that the dental professionals were too cautious and avoided challenging exposures. According to these coaches, the dental professionals did not understand the importance of exposure and did not manage to help the children challenge their fear. Furthermore, two coaches felt uncomfortable with taking the time of the dental staff for training the child. We should also mention that, in three cases, the dental clinics meeting the participants initially asked for extra payment from our clinic for the extra costs of training the participants. However, when the clinic managers learned directly about the project from us, or via the parents, they dropped their requests for extra payment. We reminded these dental clinics that four visits, a total of 105 min, is not an unreasonable amount of time to train children who suffer from dental anxiety and that treatment of children with dental anxiety is a part of pediatric dentistry. In two cases, there was concern about getting too many participants from our project. We informed these clinics that no one clinic would receive too many such requests.

## Discussion

### Principal Findings

The aim of this study was to test the hypothesis that psychologist-guided ICBT improves school-aged children’s and adolescents’ ability to manage dental anxiety by (1) decreasing avoidance and affecting the phobia diagnosis and (2) decreasing the dental fear and increasing the target groups’ self-efficacy. The study also aimed to examine the feasibility and acceptability of this novel treatment. The results show large within-group effect sizes (Cohen *d*) for the treatment, ranging from 0.9 to 2.2 for the outcome measures. Participants (8-15 years) improved their ability to cope with dental procedures, reduced their negative feelings and thoughts about dentistry, and increased their dentistry-related self-efficacy after the treatment. High levels of feasibility and acceptability (engagement, adherence, completed measures, and patient or parent satisfaction) were indicated by the results. Regarding compliance, the number of participants who completed all 12 modules was low, which indicates a need for reducing the number of modules.

Moreover, this study indicates that the treatment was acceptable from the perspective of dental health staff involved. Participating parents were able to book and attend at least three appointments at the dental clinic and meet a dental assistant, a hygienist, or a dentist. Parents overall reported high level of satisfaction concerning cooperation with the dental health staff that conducted the in vivo exposures.

### Comparisons With Previous Work

Our results are in line with previous findings from a clinical trial testing a face-to-face CBT protocol for children and adolescents with dental anxiety [14]. The observed effect sizes of face-to-face CBT, however, were larger, ranging from 1.3 to 2.9 for the outcome measures. In the face-to-face CBT trial, participants managed more dental procedures in the BAT on average, and fewer participants met the diagnostic criteria for dentistry-related specific phobia compared with the participants in this ICBT study. This result might imply that ICBT is less effective than CBT, but these differences could also be because of differences in study design between the two studies, such as the differing age ranges for participants, different versions of the primary outcome measure, different level of severity of phobia for populations in these studies, and access to treatment at general or pediatric specialist dental clinics. ICBT combined with treatment at the specialist clinic could result in better treatment effect than ICBT combined with treatment in general dentistry. Thus, the results from these two studies are not entirely comparable. Due to small group sizes, we have not been able to conduct subanalyses that allow us to study the effect of age and symptom severity on the treatment efficacy. However, a meta-analyses conducted by another research team did not find age effects in results of exposure treatment (CBT) of child and adolescent anxiety [38].

Compared with self-help CBT for children with dental anxiety [21], our treatment is more resource-demanding (patient and parents are guided by a psychologist during 12 weeks). However, the self- help resource in dentistry we mentioned earlier and the material it consists of has not been evaluated in RCT studies. The effect of self-help CBT for patients with severe symptoms and the diagnosis of dentistry-related specific phobia is uncertain. There is some evidence in the literature that guided ICBT is more effective than unguided [39]. In this study, we wanted to build the new Internet-based treatment on the methods and material of the face-to-face psychologist-guided CBT program for pediatric dental phobia, which is evaluated in a RCT study [14] and use the benefits of guided ICBT.

One interesting finding of this trial was that the effect size for children’s dentistry-related self-efficacy was as large as in the face-to-face study. This is a promising result as self-efficacy may be linked to clinically significant and sustainable behavioral change. In our earlier face-to-face CBT study, we hypothesized that differences in self-efficacy may explain why children in the treatment-as-usual group achieved, to a lesser extent, clinically significant behavior change [14]. Our results suggest that ICBT, like CBT, has the potential to affect self-efficacy. This is important as self-efficacy may be a mechanism of change in psychotherapy [40,41]. The self-efficacy effect could also be related to the film exposures included in both face-to-face therapy and ICBT. These film scenes were based on the principles of model learning and promoted processes that facilitate development of self-efficacy [40]. Studies have shown that vicarious exposure and film modeling can be as effective as or even more effective than live exposure when dealing with fear stimuli [42,43].

Another interesting finding was that the participants’ fear of injection in general (in health care settings, eg, vaccination) decreased, as indicated by the clinical interview with parents and the injection phobia scale. The effect, however, was limited to changes from baseline to posttreatment measurements and was not maintained at the follow-up assessment.

In an earlier interview study with children and adolescent who had received CBT in dentistry, parents and children expressed uncertainty about the children’s ability to manage injections outside of dental care (eg, vaccinations and blood sampling). Continued fear of injection in a health care setting could threaten improvement from CBT treatment in dentistry and enhance the risk of relapse to intraoral injection phobia [9]. Treatment module 12 contained recommendations for practices to help generalize the child’s ability to receive injections in other health care settings. However, as mentioned in the results, only 5 of the participants completed and sent back the home assignments for module 12, which could mean that the remaining participants did not follow or use these recommendations. To address this, we should perhaps place the part of treatment dealing with general injection phobia in an earlier module and stress it more by incorporating planned exposures for injection training with health care staff.

The results of this study also agree with evaluations of evidence-based psychological treatments such as one-session CBT treatment for pediatric-specific phobias. In a study of one-session treatment, 49% of participants were free of a specific-phobia diagnosis at a 6-month follow-up [44] compared with 53% at the 1-year follow-up in our study. Others have reported similar results for ICBT in children with specific phobias, with approximately 50% of children at a 3-month follow-up free from a specific-phobia diagnosis [18,20].

Comparing one-session treatment and therapist-guided Internet treatment for spider phobia, both groups achieved clinically significant results according to the BAT. Approximately 70% achieved this effect at a 1-year follow-up [19]. Similar effects appear for ICBT in children with anxiety disorder, with 75% of participants reported as diagnosis-free at a 6-month follow-up [17]. The two booster sessions that participants were offered after treatment and before the follow-up may explain the higher percentage of diagnosis-free participants in the last study [20]. Implementing booster sessions in ICBT for pediatric dental anxiety may be a way to increase its treatment efficacy.

### Limitations

This study is limited by its design and lack of a control group, which would make between-group comparisons possible. Participant improvements could hypothetically be a result of other factors than the treatment, such as time and maturation of the child; thus, the results should be interpreted with caution. However, CBT for specific phobias has shown good effects in numerous controlled studies [11,44]. On average, the participants in this study reported suffering from dental anxiety for 4 years before the study, which contradicts the supposition that this fear would disappear without treatment.

To take part in this study, a parent or coach needed to have the ability to manage the project information, apply for participation, and continuously support the participant during the project. Participants whose coach lacked these resources and abilities would have difficulty participating. As in several other ICBT studies, a high percentage of the coaches were well-educated, which may imply a limitation that parents with restricted resources and low education would struggle to access treatment.

Another limitation is that some of the measures used in this study were new and constructed in our research group. We had to construct these instruments because of a lack of suitable instruments for measuring behavioral and cognitive dimensions of pediatric dental phobia in the literature. These new measures need to be validated in larger studies.

### Future Directions

Strengths of this study include a threefold measurement (child, coach, and psychologist assessments). Furthermore, the trial was conducted in a naturalistic dental care setting, which means that participants had the opportunity to experience exposure in vivo. Nevertheless, there is a need to modify and customize ICBT for participants and coaches who are less accustomed to reading texts, for instance by including short filmed lectures and fewer modules. Using ICBT to facilitate interaction between children or adolescents, coach or parent, and the medical or dental care staff is a novel approach implemented in this study. There is a potential to further develop this approach by increasing interaction with dental care staff. In this study, coaches brought short information sheets about ICBT to the staff they met. Providing access to brief ICBT courses for the dental care staff and supervision for them could increase treatment effect.

Another way to increase the dental health staff´s interaction could be to give them access to the Internet platform for the ICBT and allow the staff to take part; comment and give feedback to the participants during the whole ICBT. Although this may make the treatment more efficacious, there is a risk for making the treatment too demanding and time-consuming for the participating staff, which could decrease the staffs’ acceptability. Studies that investigate the dental health staff acceptability for ICBT by qualitative interviews are important to conduct. Future studies should also test the ICBT for pediatric dental anxiety in RCTs.

### Conclusions

If results of this open trial can be replicated in future controlled studies, there would be a potential for dental care systems to gain an effective evidence-based psychological treatment for children with dental anxiety—regardless of where they live or their access to specialist pediatric dental care —with relatively low personnel costs. While acknowledging the limitations of this study, we conclude that ICBT for dental anxiety in children and adolescents could be a feasible and efficacious treatment with the potential to increase accessibility to effective treatment.

## Abbreviations

BAT: behavior avoidance test
CBT: cognitive behavioral therapy
CFSS-DS: Children's Fear Survey Schedule-Dental Subscale
CNCD: children’s negative cognitions in dentistry
DAWBA: Development and Well-Being Assessment
DSM-IV: Diagnostic and Statistical Manual of Mental Disorders-4th edition
ICBT: Internet-based cognitive behavioral therapy
IPSC: injection phobia scale for children
K-SADS-PL: Kiddie-Schedule for Affective Disorders and Schizophrenia for School-Age Children-Present and Lifetime version
PG-BAT: picture-guided behavior avoidance test
P-SEQ-DA: Parental Self-Efficacy Questionnaire for Dental Anxiety
RCT: randomized controlled trial
SEQ-SP: Self-Efficacy Questionnaire for Specific Phobias
SD: standard deviation
VAS: visual analog scale
